# Evaluation of Safety and Potency of Kyasanur Forest Disease (KFD) Vaccine Inactivated with Different Concentrations of Formalin and Comparative Evaluation of In Vitro and In Vivo Methods of Virus Titration in KFD Vaccine

**DOI:** 10.3390/biomedicines11071871

**Published:** 2023-06-30

**Authors:** Ullas Gowda K. Srikanth, Chandranaik B. Marinaik, Amitha Reena Gomes, Doddamane Rathnamma, Sonnahallipura M. Byregowda, Shrikrishna Isloor, Archana Munivenkatarayappa, Mudalagiri D. Venkatesha, Suguna Rao, Apsana Rizwan, Raveendra Hegde

**Affiliations:** 1 Institute of Animal Health and Veterinary Biologicals, Karnataka Veterinary, Animal and Fisheries Sciences University—KVAFSU, Bangalore 560 0624, India; drullasgowdaks@gmail.com (U.G.K.S.); amithagomesvet@gmail.com (A.R.G.); smbyregowda@gmail.com (S.M.B.); sahiahvb@gmail.com (A.M.); mdvenkatesha@gmail.com (M.D.V.); drapsana@gmail.com (A.R.); honkankereravi@yahoo.com (R.H.); 2 Veterinary College, Karnataka Veterinary, Animal and Fisheries Sciences University—KVAFSU, Bangalore 560 0624, India; rathnarohit@gmail.com (D.R.); kisloor@gmail.com (S.I.); sugunabg@gmail.com (S.R.)

**Keywords:** kyasanur forest disease, kyasanur forest disease virus, KFD, KFD vaccine, formalin, real-time PCR

## Abstract

We evaluated the safety and potency of the Kyasanur Forest disease (KFD) vaccine inactivated with different formalin concentrations in mice, since the side effects due to higher formalin concentrations have been a major reason for vaccine refusal. Furthermore, with an objective to reduce the use of mice in vaccine testing, we performed quantification of the KFD virus by real-time PCR and compared it with in vivo titration in mice. The KFD vaccine prepared in chicken embryo fibroblast cells was inactivated with 0.04%, 0.06%, and 0.08% concentrations of formalin. The vaccine inactivated with 0.04% and 0.06% formalin failed the safety test, whereas the KFD vaccine inactivated with 0.08% formalin was safe and potent with a log protective index of 5678 in mice. This reduced formalin content may induce no/lesser side-effects of pain/swelling which may increase the vaccine acceptance. The real-time PCR on individual KFD vaccine harvests interpreted that when the CT value of each harvest is <20, the vaccine will have sufficient viral particles to pass the potency test. Comparison of the real-time PCR on tenfold dilutions of the pooled harvests with in vivo mice inoculation test revealed that the 1MLD_50_ of the vaccine lies in the tenfold dilution that yields CT values between 31 and 34.

## 1. Introduction

Kyasanur Forest disease (KFD) is an emerging vector-borne viral zoonosis caused by the Kyasanur Forest disease virus (KFDV). The KFDV belongs to the family *Flaviviridae* and the genus *Flavivirus*, which comprises a number of mosquito- and tick-borne viruses. The virus was discovered in 1957 during an outbreak in monkeys that spread to people in the Kyasanur Forest of Karnataka’s Shivamogga district in India [1]. The KFDV is transmitted to humans and monkeys predominantly by blood–meal absorption by KFDV-infected ticks of *Haemaphysalis* species [2], indicating that the virus is arthropod-borne. The virus is maintained in these forest environments by cycling with reservoir species such as rats, shrews, bats [3], and probably cattle [4,5]. Humans may contract the virus by handling and interacting with diseased or dead monkeys, as villagers access forest areas to establish rice crops, and pasture cattle, and to collect firewood and leaves [2]. During these activities, people come in close contact with infected ticks and possibly diseased or dead monkeys in forests [6]. Furthermore, climatic changes, increased deforestation, and agricultural activities have resulted in additional contact between ticks, monkeys, and humans in KFD-endemic areas [7].

The course of the disease can vary from subclinical infection to fatal cases with hemorrhagic complications, but the pathology of KFDV remains incompletely understood. The disease has a case fatality rate of 3–5% and there are no approved antivirals. Vaccination of susceptible populations in the disease endemic area is the most effective measure for control of the KFD. The only existing licensed vaccine is a formalin-inactivated tissue culture vaccine. The Institute of Animal Health and Veterinary Biologicals (IAH and VB) Bengaluru is producing the cell culture-based KFD vaccine and is supplying it to susceptible people in KFD disease-endemic regions of India, for the past 23 years. The IAH and VB is the only institute in the country that produces vaccines against KFD. Vaccination regimes include two doses of vaccine administered at an interval of one month, by s/c route to persons aged from 7–65 years, since the immunity conferred by the vaccine is short-lived [8] booster doses are recommended within 6–9 months of the primary vaccination. The effectiveness of the vaccine was 62.4% among those who received two doses and 82.9% for those who received an additional booster dose as compared to the unvaccinated individuals [9]. The vaccine and vaccination program have significantly helped people and have brought down the disease intensity, morbidity, suffering, and mortality in the past 50 years.

The vaccine that is currently available is a 0.1% formalin inactivated tissue culture vaccine. The vaccine, though effective, has mild side effects of swelling and irritation at the site of injection caused by the formalin, and this has been a primary reason for vaccine refusal by local people in disease-endemic regions [8,9]. There is a growing demand from the government and also the general public to reduce the formalin concentration in the vaccine. In view of this limitation, there has been a growing demand to reduce the formalin concentration used for inactivating the KFD virus in the vaccine, without affecting its safety and potency. Considering these field-oriented limitations of the available KFD vaccine, the current work was taken up with the objectives of evaluating the safety and efficacy of the Kyasanur Forest disease (KFD) vaccine inactivated with different concentrations of formalin in mice. Furthermore, the study employed the TaqMan real-time PCR for titration of the KFDV in the vaccine and compared it with the currently used in vivo mice inoculation tests, to examine if we can reduce the use of laboratory animals in quality testing of vaccines in the future.

## 2. Materials and Methods

The KFD vaccine used in this study was prepared by growing the KFD seed virus strain P9605 (GenBank Accession Number. JF416958; passaged in mouse brain (passage 4) with titer (MLD_50_) of 10^7^/0.2 mL) in chick embryo fibroblast primary culture. The KFD seed virus was procured from the National Institute of Virology (NIV), Pune, India.

### 2.1. Procedure for Chick Embryo Fibroblast Monolayer Culture

Primary chick embryo fibroblast (CEF) culture was prepared from 9-day-old chick embryos as per standard protocols [10,11]. Briefly, the procedure included the CEF monolayers in each Roux flask being infected with 0.01 multiplicity of infection (MoI) of KFDV by the adsorption method at 37 °C for 1 h. The Roux flasks were incubated at 37 ± 1 °C for 48 h. The first harvest (H1) of the cell culture supernatant was performed at 48 h post-infection and the subsequent four harvests (H2, H3, H4, and H5) were performed at every 24 h interval.

### 2.2. Inactivation of the KFD Virus

Based on the quantity of the cell culture supernatant harvests collected, formalin was added such that the final concentration of formalin was 0.08%, 0.06%, or 0.04% as required for this study. The containers with the cell culture harvest with KFD virus added with formalin were kept in a shaker incubator at 4 °C for 14 days. The inactivated KFD vaccine was clarified by passing through a microfiltration unit comprising different filter cassettes of 0.8 µ, 0.6 µ, and lastly through a 0.2 µ filter.

### 2.3. Safety and Potency Test

Safety and potency tests for the vaccine were carried out as per the procedure outlined by the National Institute of Virology, Pune, India [10,11].

#### 2.3.1. Safety Test Procedure

The KFD vaccine inactivated with different concentrations of formalin was inoculated to two groups of mice, 2–3-day-old suckling mice (*n* = 20 for each concentration of formalin) and 3–4-week-old subadult mice (*n* = 20 for each concentration of formalin), by intra-cerebral (i/c.) and subcutaneous (s/c.) routes. Each suckling mouse received 0.02 mL i/c and 0.03 mL s/c and the subadult mouse received 0.03 mL i/c and 0.5 mL s/c. Inoculated mice were observed for 21 days for sickness. Mice found dead within 48 h were discarded and considered as nonspecific.

The safety test procedure was performed for all three (0.04%, 0.06%, 0.08%) concentrations of formalin inactivated vaccines. The vaccine with the lowest concentration of formalin that passed the safety test was subjected to a potency test.

#### 2.3.2. Potency Test Procedure

Potency test was carried out in two groups of mice, the vaccinated/immunized group and the control group, each group having 40 and 50 mice, respectively. For this test, 3–4-week-old mice were selected. The vaccine was diluted at 1:16 in chilled GMEM. Forty mice were inoculated with 0.5 mL of 1:16 diluted vaccine by intra peritoneal (i/p) route on day 0 and day 7. Fifty mice of the same age (matched controls) were left unvaccinated. On day 14 of the post-first dose of vaccination, the challenge KFD virus was titrated in immunized and control mice by inoculating tenfold serial virus dilutions in a group of ten mice, each mouse received 0.2 mL of KFD virus, by i/p. The dilutions of the challenge KFD virus inoculated in immunized mice were 10^−2^ to 10^−5^, whereas, in the control group, it was 10^−5^ to 10^−9^.

The log MLD_50_ titers (the reciprocal of the highest dilution of virus that causes mortality in 50% of the inoculated mice) of the virus in the vaccinated and control group were calculated by the Reed and Muench method [12]. The difference in logarithmic titers of control and immunized mice was the log protective index.

### 2.4. Comparison of In Vitro and In Vivo Tests for Titration of Non-Cytopathic KDFV in the Vaccine

#### 2.4.1. Titration of Virus in Vaccine for MLD_50_

Virus titration (MLD_50_) was performed on the pooled cell culture supernatant (vaccine harvests) collected after infection with the P-9605 KFDV strain (before adding formalin).

The cell culture supernatants (pre-inactivated) collected at each harvest were pooled (H1 + H2 + H3 + H4 + H5). We prepared serial tenfold dilutions of the pooled virus in glass vials (0.5 mL virus + 4.50 mL maintenance medium) from 10^−1^ to 10^−8^. Five mice (3–4 weeks old) were injected intracerebrally with 0.03 mL each of the dilutions starting from 10^−1^ to 10^−8^.

Plain media was injected intracerebrally into five mice as the control group. Another five mice were injected with undiluted cell culture supernatant as the positive control.

All mice were observed daily for 14 days for any symptoms and death. We recorded the number of normal animals, and sick animals showing symptoms of tremors, paralysis, prostration, or death. The titer of the virus was calculated by the Reed and Muench formula and expressed as MLD_50_/0.03 mL of the pre-inactivated vaccine harvest.

#### 2.4.2. Quantification of KFDV by Real-Time, Reverse Transcriptase Polymerase Chain Reaction

Quantification of KFDV in individual vaccine harvests (H1, H2, H3, H4, and H5) as well as in pooled harvests (H1 + H2 + H3 + H4 + H5) was performed by in vitro test real-time, reverse transcriptase polymerase chain reaction (real-time RT-PCR). The same tenfold serial dilutions (10^−1^, 10^−2^, 10^−3^, 10^−4^, 10^−5^, 10^−6^, 10^−7^, and 10^−8^) of the vaccine harvest that were used for virus titration in mice (described under Section 2.4.1), were used for quantification of the virus by real-time PCR.

RNA was extracted from individual harvests and from each of the tenfold dilutions of the pooled harvest as per the procedure described in QIAamp^®^ Viral RNA Extraction Kit procured from M/s Qiagen. The RNA extracted from diluted KFD seed virus was used as the positive control and RNA extracted from classical swine fever virus (a member of the virus family *Flaviviridae)* was used as the negative control. The extracted RNA was subjected to real-time PCR as per the procedure described by Mourya and coworkers [13] in a QIAGEN Rotor-Gene Q thermal cycler. The primers and the probe used for the study included Forward primer 5’ TGGAAGCCTGGCTGAAAGAG 3’ Reverse primer 5 TCATCCCCACTGACCAGCAT 3’ and the TaqMan probe 5’ ATGGAGAGGAGCGCCTGACCCG 3’. The PCR was run with one cycle of reverse transcription at 50 °C for 30 min and Taq inhibitor inactivation at 95 °C for 10 min followed by 40 repeated cycles of 95 °C for 15 s and 60 °C for 1 min, amplifying a specific fragment of 63 bp amplicon on NS gene of KFDV. At the end of 40 cycles of amplification, the results were recorded for individual harvests and serially tenfold diluted pooled harvests.

### 2.5. Regulatory Permission to Conduct Research on KFD

Permissions of the Institutional Animal Ethics Committee (IAEC) for laboratory animal experiments in mice at the ‘KFD vaccine production laboratory’ of the Institute of Animal Health and Veterinary Biologicals, Bengaluru was accorded vide order, IAEC.CODE: IAH/IAEC/BP/PE/KFD/2021-22/13, dated 26 August 2021. The Institutional Biosafety Committee (IBSC) permission was accorded to conduct this research work vide order IAH:IBSC: Project number-07-08/21, dated 26 August 2021.

## 3. Results

Evaluation of the safety and potency of the Kyasanur Forest Disease (KFD) vaccine inactivated with different concentrations of formalin in mice.

### 3.1. Preparation of Kyasanur Forest Disease (KFD) Vaccine in Chick Embryo Fibroblast (CEF) Primary Culture

The Kyasanur Forest disease virus is well propagated in the chick embryo fibroblast monolayer culture. Since the KFD virus was noncytopathic in the CEF indicator system there were no changes in cell morphology and monolayers in both infected and un-inoculated controls which remained intact even after 144 h of infection at the fifth harvest. At each harvest, cell culture supernatant was inactivated using 0.04%, 0.06%, and 0.08% formalin.

### 3.2. Safety Test KFD Vaccine in Mice

The safety of the KFD vaccine inactivated with different concentrations of formalin was tested by giving the vaccine via intracerebral and subcutaneous routes as previously described.

#### 3.2.1. Safety Test of 0.04% Formalin Inactivated KFD Vaccine in Mice

**Figure 1:**
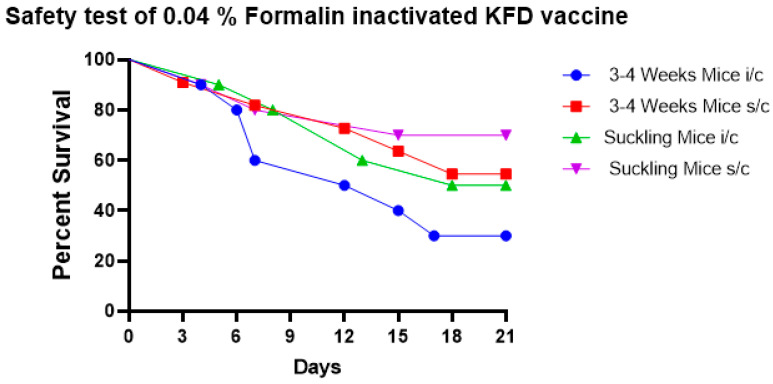
The safety test of 0.04% formalin-inactivated KFD vaccine in mice shows that 0.04% formalin fails to completely inactivate the KFD virus in the vaccine as noticed by mortality in 3–4-week-old mice and in suckling mice when the vaccine was inoculated by s/c and i/c routes.

The results showed that 0.04% formalin fails to completely inactivate the KFD virus in the vaccine as evidenced by KFD symptoms of ruffled hair coat, hunched back appearance, weakness, paralysis, and death in subadult and suckling mice. The survival percentage of mice inoculated with the 0.04% formalin-inactivated KFD vaccine is depicted in Figure 1.

#### 3.2.2. Safety Test of 0.06% Formalin Inactivated KFD Vaccine in Mice

**Figure 2:**
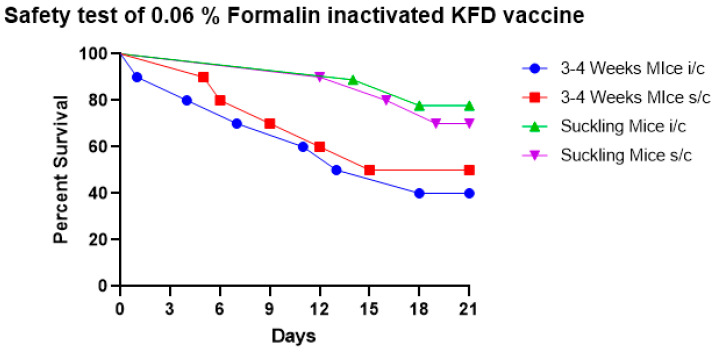
The safety test of 0.06% formalin-inactivated KFD vaccine in mice shows that 0.06% formalin fails to completely inactivate the KFD virus in the vaccine as noticed by mortality in 3–4-week-old mice and in suckling mice when the vaccine was inoculated by s/c and i/c routes.

The results showed that 0.06% formalin fails to completely inactivate the KFD virus in the vaccine as evidenced by KFD symptoms of ruffled hair coat, hunched back appearance, weakness, paralysis, and death in sub-adult and suckling mice. The survival percent of mice inoculated with the 0.06% formalin-inactivated KFD vaccine is depicted in Figure 2.

#### 3.2.3. Safety Test of 0.08% Formalin Inactivated KFD Vaccine in Mice

**Figure 3:**
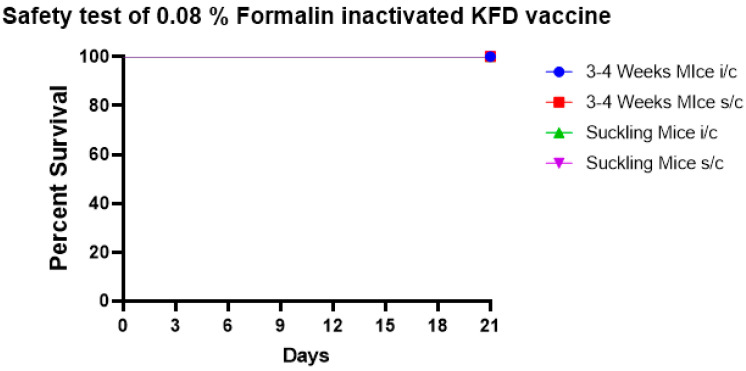
The safety test of 0.08% formalin-inactivated KFD vaccine in mice shows that 0.08% formalin completely inactivates the virus in the vaccine and the vaccine is safe in 3–4-week-old mice and in suckling mice when inoculated by s/c and i/c routes with 100% survival.

The results showed that at the end of the 21st day all mice, both subadult mice and suckling mice, were healthy, in both i/c and s/c routes of inoculation (Figure 3).

### 3.3. Potency Test for 0.08% Formalin Inactivated KFD Vaccine

**Figure 4:**
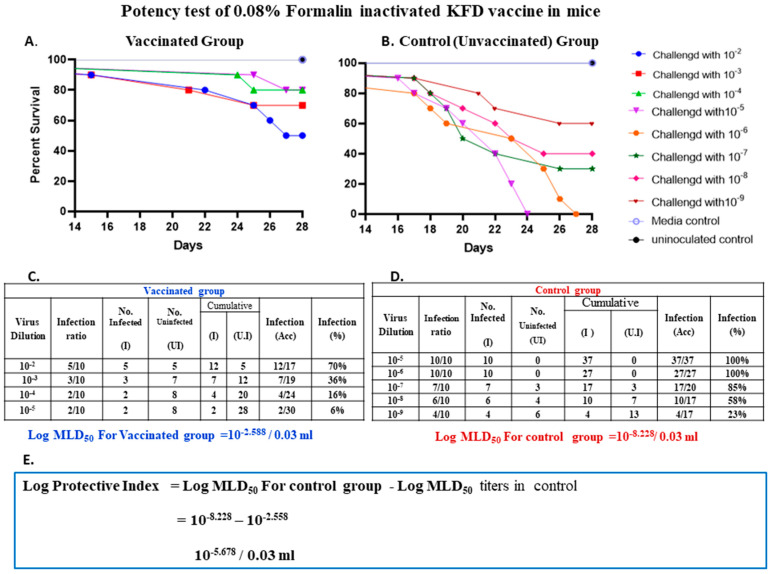
Potency test of 0.08% formalin inactivated KFD vaccine in mice. (**A**) 3–4-week-old mice were inoculated with 0.5 mL of vaccine by i/p route on days zero and seven. On day 14 of the post-first dose of vaccination, the challenge virus dilutions of 10^−2^ to 10^−5^ were inoculated and observed for fourteen days. The figure gives the mortality pattern and survival percent of challenged vaccinated mice. (**B**) Age-matched controls were left unvaccinated (control) and were challenged with virus dilutions of 10^−5^ to 10^−9^ and were observed for 14 days. The figure gives the mortality pattern and survival percent of challenged unvaccinated mice. Media-only control (*n* = 10) and uninoculated control (*n* = 10) were also maintained for the potency test. (**C**) When enumerated using the Reed and Muench method, results showed that the 0.08% formalin-inactivated KFD vaccine was able to protect 30% of mice in the group given 10^−2^ dilutions of the virus; protected 64% of mice in the group given 10^−3^ dilutions and protected 84%, and 94% of mice given 10^−4^ and 10^−5^ dilution of the challenge virus, respectively. The logarithmic titer of the virus in the vaccinated group was 10^−2.558^. (**D**) In unvaccinated control mice, by the end of the potency test, all mice inoculated with 10^−5^ and 10^−6^ dilutions of the virus died with KFD symptoms, whereas 85%, 58%, and 23% of mice died in groups given with virus dilution of 10^−7^, 10^−8^ and 10^−9^, respectively. The logarithmic titer of the virus in the unvaccinated control mice group was 10^−8.228^. (**E**) The log protective index, defined as the difference in logarithmic titer MLD_50_ in control mice and the vaccinated mice, was 10^−5.678^.

A potency test was carried out in two groups of mice, vaccinated/immunized and control group, each group having 40 and 50 mice, respectively. For this test, 3–4-week-old mice were selected, and the vaccine was diluted 1:16 in chilled MEM. Forty mice were inoculated with 0.5 mL of diluted vaccine by intra-peritoneal (i/p) route on days zero and seven. Fifty mice of the same age-matched controls were left unvaccinated. On day 14 of the post-first dose of vaccination, the challenge virus was titrated in immunized and control mice by inoculating tenfold serial dilutions of the virus in a group of ten mice, each mouse receiving 0.2 mL i/p. The dilutions of the challenge virus inoculated in immunized mice were 10^−2^ to 10^−5^, whereas, in the control group it was 10^−5^ to 10^−9^. The detailed result of the potency test is depicted in Figure 4A,B.

Results when enumerated using the Reed and Muench method, showed that the 0.08% formalin-inactivated vaccine was able to protect 30% of mice in the group given 10^−2^ dilutions of the virus; the vaccine protected 64% of mice in the group given 10^−3^ dilutions of the challenge virus, and protected 84% and 94% of mice given 10^−4^ and 10^−5^ dilution of the challenge virus, respectively (Figure 4A,C).

In unvaccinated control mice, by the end of the potency test, all mice inoculated with 10^−5^, and 10^−6^ dilutions of the virus died with KFD symptoms, whereas 85%, 58%, and 23% of mice died in groups given virus dilution of 10^−7^, 10^−8^, and 10^−9^, respectively (Figure 4B,D).

The proportional distance (PD) is the actual endpoint dilution (the dilution which would give an exact 50% mortality) in mice which was calculated by the Reed and Muench formula [12].

$$Proportional\;Distance\left(PD\right)=\frac{Per\;cent\;mortality\;next\;above\;50\%–50}{Per\;cent\;mortality\;next\;above\;50\%–Per\;cent\;mortality\;next\;below\;50\%}$$

The log MLD_50_ of KFD virus in immunized and control mice was calculated by the Reed and Muench formula [12].

Log MLD_50_ = Log Dilution next above 50% + (PD × Log Dilution factor)

Log protective index = Difference in logarithmic titers of control and immunized mice = 10^−8.228^–10^−2.558^ = 10^−5.678^/0.03 mL

The logarithmic titer of the virus in the vaccinated group was 10^−2.558^ (Figure 4C) and 10^−8.228^ in the unvaccinated control mice. (Figure 4D). As per NIV guidelines, the log protective index is defined as the difference in logarithmic titer MLD_50_ in control mice and the vaccinated mice. In this study, the log protective index was 10^−5.678^ (Figure 4E). As per the NIV Pune protocol, if the vaccine used is diluted at 1:16 dilution, the log protective index should be 5.4 and above. Hence, with a log protective index of the 0.08% formalin inactivated KFD vaccine passed the potency test.

### 3.4. Quantification of Kyasanur Forest Disease Virus by Real-Time PCR and Its Comparison with In Vivo Titration in Mice

The KFDV used for vaccine production is a noncytopathic virus, so titration of the virus requires in vivo models such as mice. This study employed real-time PCR to quantify the virus in the vaccine and compared it with in vivo mice tests.

### 3.5. In Vivo Quantification of Virus by Mice Inoculation Test

**Figure 5:**
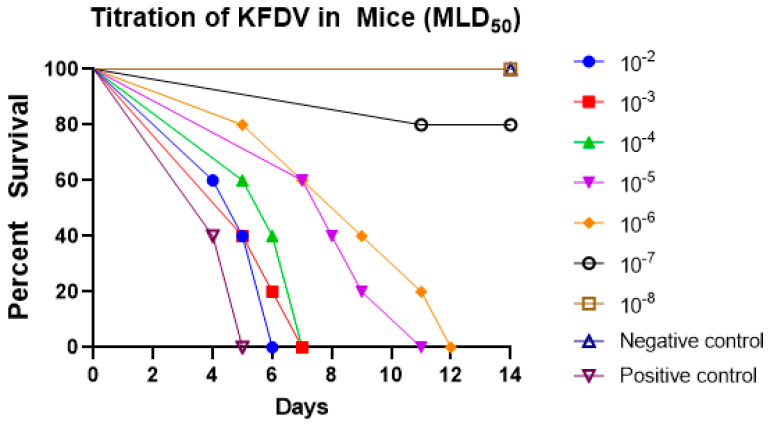
In vivo titration of KFDV (MLD_50_) by mice inoculation test.

The aliquots of pre-inactivated cell culture supernatant of all the harvests (H1, H2, H3, H4, and H5) were pooled and prepared serial tenfold dilutions of the pooled virus in 5 mL glass vials (0.5 mL pooled harvest + 4.5 mL chilled maintenance medium) from 10^−1^ to 10^−8^. We injected five mice of 3–4 weeks old, intracerebrally, with 0.02 mL of each dilution starting from 10^−1^ to 10^−8^. Plain media was injected intracerebrally into a group of five mice as the control group and another five mice were injected with the KFD seed virus as the positive control group. Mice were observed daily for 14 days for symptoms and deaths (Figure 5).

At the end of the 14th day, all the mice of virus dilutions at 10^−2^, 10^−3^, 10^−4^, 10^−5^, and 10^−6^ showed symptoms/sickness or death. One mouse died from virus dilution 10^−7^ and all mice were healthy from virus dilution 10^−8^ (Figure 5).

Computation of the results by the Reed and Muench method showed that the KFDV in pre-inactivated vaccine dilutions 10^−2^, 10^−3^, 10^−4^, 10^−5^, and 10^−6^ infected 100% of mice, whereas 10^−7^ infected 20% of mice and 10^−8^ virus did not cause infection in any mice. Thus, the titer MLD_50_ of the KFDV in pre-inactivated vaccine was 10^−6.375^/0.03 mL.

### 3.6. Quantification of KFDV Titre by Real-Time PCR

The real-time PCR was performed on RNA extracted from individual harvests (H1, H2, H3, H4, and H5) and also on serially tenfold diluted pooled harvests (H1 + H2 + H3 + H4 + H5).

The real-time PCR results of individual harvests (H1, H2, H3, H4, and H5) depict that H1 has a CT value of 15.59, H2 has a CT value of 18.06, H3 has a CT value of 17.3, H4 has a CT value of 19.31, and H5 has a CT value of 19.7. The positive control had a CT value of 28.34.

**Figure 6:**
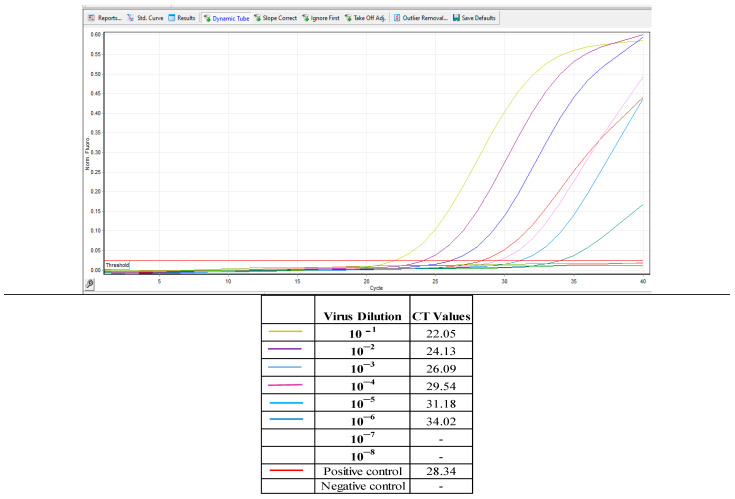
Real-time RT-PCR CT values of tenfold dilutions of pooled harvest.

The real-time PCR results (CT value) of the tenfold diluted pre-inactivated KFDV vaccine (Figure 6) yielded a CT value of 22.05 in 10^−1^, 24.13 in 10^−2^, 26.09 in 10^−3^, 29.54 in 10^−4^, 31.18 in 10^−5^, and 34.02 in 10^−6^. The dilutions of 10^−7^ and 10^−8^ did not yield CT values after 40 cycles of amplification (Figure 6).

Virus dilutions from 10^−1^ to 10^−6^ showed 100% infectivity in in vivo titration in mice and also observed the amplification in real-time PCR. In the virus dilution of 10^−7^, 20% infectivity was observed in mice, but real-time PCR did not yield positive results after 40 cycles of amplification in (Table 1).

## 4. Discussion

Demonstration of mosquito transmission of yellow fever from a patient to human volunteers and isolation of the Yellow Fever virus in Rhesus monkeys [14] and the development of a protective vaccination procedure for the Yellow Fever virus in the early 1930s paved a major path to study arthropod-borne viruses.

The scientists from the USSR isolated the Russian Spring-Summer Encephalitis (RSSEV) virus (now called the Tick-Borne Encephalitis virus (TBEV), far eastern subtype) the first tick-borne agent of severe consequence to humans and were able to develop an effective formalized mouse brain vaccine for RSSEV in 1939 [15]. This became the key to studies on the “Sylvian circulation cycle” of tick-borne viruses which subsequently provided the pattern for initial studies of related viruses. Taking clues from these studies, Indian scientists developed the 0.1% formalin-inactivated tissue culture KFD vaccine in the 1960s [10,16]. This vaccine has been extensively used as prophylaxis in KFD-endemic regions.

The vaccine produced at IAH and VB, Bengaluru since 2000 has helped in significantly reducing the morbidity from 2000–2005 (1832 cases and 38 deaths) to 2016–2020 (787 cases and six deaths) [17] compared to the periods where vaccination was not practiced (for example between the period of 1983 to 1990 a total of 1930 cases and 179 deaths were attributed to KFD in the Karnataka state). However, formalin-associated side effects at the site of vaccine injection have been a major reason for vaccine refusal in rural setups [8,9].

Serious hemorrhagic and neurological manifestations of KFD have been observed mainly in poor villagers in the KFD-endemic areas compared to the laboratory personnel who practice better hygiene and better nourishment [18]. Chronic diseases such as tuberculosis and helminthic infection have been shown, though indirectly, to be associated with serious consequences of KFD [19]. Under these situations it is important to increase the vaccination coverage in rural areas and forest areas where poor people reside and are most vulnerable to KFD; unfortunately, no serious attempts have been made in the past fifty years to improve the currently available vaccine both in terms of improving its quality and in reducing the side effects of the vaccine.

We used 0.04%, 0.06%, and 0.08% formalin inactivated KFD vaccines. The 0.04% and 0.06% formalin inactivated KFD vaccines did not completely inactivate the virus and failed the safety test, whereas the 0.08% formalin inactivated KFD vaccine passed the safety test. This could be because the process of inactivation using formalin depends on the duration of inactivation, the temperature of inactivation, and the quantity of virus in the cell culture harvest [20]. The temperature and duration used in this study were possibly not sufficient for inactivating all the virus particles in the 0.04% and 0.06% formalin inactivated vaccines. The results in this study indicate that 0.08% formalin will completely inactivate all the KFD virus particles at 4 °C for 14 days, hence it was safe in mice and passed the safety test as there were no diseases or sickness in all mice inoculated with the vaccine inactivated with 0.08% formalin and the findings were in accordance with the works of Dandawate et al. [10,21] who recorded similar findings for a 0.1% formalin-inactivated KFD vaccine. This vaccine was further subjected to potency tests in mice.

The potency test showed that the 0.08% formalin inactivated KFD vaccine retained the immunogenicity and is potent enough to protect against the challenge virus with a protective index of 5.678 which is above the required index value to pass the potency test.

The results of the potency test of the 0.08% formalin-inactivated KFD vaccine were comparable with the potency test results achieved by Dandawate et al., (1980) [21] who developed the first KFD vaccine for human use which had the log protective index of 5.4 when used at a dilution of 1:16. Furthermore, the log protective index value of the 0.08% formalin-inactivated KFD vaccine was comparable to the potency of the existing 0.1% formalin-inactivated vaccine [22].

Formalin has been used as an inactivating agent in many human vaccines, viz., the West Nile fever virus disease (WNFV) vaccine has 0.2% of formalin as an inactivating agent [23]; likewise, in the Japanese Encephalitis virus (JEV) vaccine, 0.05% of formalin is used for the virus inactivation process [24]; and for the Influenza vaccine, 0.02% of formalin is used for inactivating the virus [25]. 

Since the concentration of formalin used is relatively lower in the 0.08% formalin inactivated KFD vaccine, compared to the currently available 0.1% formalin inactivated KFD vaccine, in all probability, this should induce less reaction or no reaction of pain and swelling at the site of injection, in humans and which may increase the vaccine coverage due to no, or fewer, side effects.

Results of molecular epidemiology studies have suggested that tick-borne Flaviviruses have evolved slowly compared to rapidly evolving mosquito-borne Flaviviruses, many of which are transported for long distances by migratory birds, persons, animals, or mosquito eggs. A comparison of available sequences of 48 KFDV isolates from India collected over a period of the last five decades showed a low level of diversity among these isolates. A maximum of 1.2% nucleotide and 0.5% amino acid difference were seen in these isolates [26]. The sequence of the 1957 KFDV reference strain (P-9605) from India and strain 651521 isolated from NIV laboratory staff members in 1965 were identical, despite their eight years of separation. The phylogeographic study of KFDV in India between 1957and 2017 has evidenced minimal adaptive evolution at site 123A/T located in the vicinity of the envelope protein [27], which plays a major role in the infection of cells and also in the protective immunity against KFDV.

Considering the recent molecular epidemiological data, the data showing that the number of KFD cases and deaths has drastically reduced from the 1980s to 2020, and the rigorous vaccination campaigns in endemic areas, it is suggested that the vaccine is quite effective, and the seed virus used for vaccine production is still relevant at the ground level. Hence the reduction in formalin concentration to 0.08% in the vaccine, and possible reduction of side effects due to formalin content in the vaccine, will further add up and aid the future prevention and control of KFD disease in endemic areas before a new generation vaccine for KFD is made available.

The KFD vaccine manufacturing involves a series of quality control assays using laboratory animals since the seed virus used is noncytopathic in chick embryo fibroblast cells used for vaccine production. The quality control tests that need to be carried out at various steps during vaccine production demand the use of a large number of laboratory animals. In this study, we applied real-time quantitative PCR for quantifying the virus in individual vaccine harvests, and also in the pooled harvests. The real-time PCR results were compared with conventional mice inoculation assays. This study intends to minimize the use of laboratory animals during the KFD vaccine production process.

Real-time PCR was applied on the KFD vaccine in this study that passed the potency test, with a required log protective index of more than 5.4 as per the NIV protocol.

Real-time PCR on individual harvests showed that the first harvest (H1) had a CT value of 15.59 followed by CT values of 18.06, 17.3, 19.31, and 19.7 for vaccine harvests H2, H3, H4, and H5, respectively.

When we put together the potency test of the vaccine under this study and CT values of individual vaccine harvests of the same vaccine, it clearly indicates that, when the CT value of individual harvests (H1 to H5) is below 20, the vaccine will have sufficient KFD virus particles to pass the potency test and protect against the KFD virus challenge.

When the tenfold dilutions of the pooled tissue culture vaccine harvests were subjected to real-time PCR, the CT values of 10^−1^ dilution was 22.05, followed by CT values of 24.13, 26.09, 29.34, 31.18, 34.02, 0, and 0 for dilutions 10^−2^, 10^−3^, 10^−4^, 10^−5^,10^−6^, 10^−7^, and 10^−8^, respectively.

The same tenfold dilutions when titrated in mice showed that the virus load in dilutions from 10^−2^ up to 10^−6^ was high and was sufficient to cause 100% mortality in all the mice in respective dilutions. The MLD_50_ of the virus was 10^−6.375^ in conventional mice inoculation.

Taking together the real-time PCR results of the tenfold dilution of pooled harvests and mice inoculation of the dilutions indicates, that when the vaccine virus is serially diluted in a tenfold ratio, one MLD_50_ of the KFD virus falls in the virus dilutions that yields CT values between 31 and 34.

The major drawback in qRT–PCR, is that it can detect the whole virus (complete virion capable of infecting) as well as only the RNA of the virus (even when it is unable to cause the infection), but in conventional mice, inoculation tests results are only based on the total virus (infective virion) that can cause the infection/symptoms and death.

However, these results can be used, and we can employ real-time PCR for quantification of the virus in the intermediate stages of the vaccine production process, and the real-time PCR results can definitely give an indication of virus load; and final potency test of the vaccine can be performed in mice, only if the real-time PCR of individual harvests have CT value of less than 20. This will save hundreds of mice that are used for the quantification of KFDV during the intermediate stages of vaccine production.

TaqMan probe-based real-time PCR is a robust technique that amplifies a specific region of the target nucleic acid. The probe acts as a third primer adding to the sensitivity of the assay [28]. The nonstructural gene 5 (NS5) is one of the most conserved genes among the members of the *Flavivirus* [13] and hence primers and probe sequence targeting the amplification of 63 bp specific region on NS5 gene was used for real-time PCR quantification of the KFDV in vaccine harvests.

Though the TaqMan real-time PCR has been used as a diagnostic method for specific identification of KFDV [13,29]. This is the first study employing TaqMan probe-based real-time PCR for the quantification of KFDV in vaccines. However, in vitro methods have been used to quantify virus load in other non-cytopathic virus vaccines; Lourenc et al. [30] used TaqMan quantitative PCR to quantify rabies virus in cell culture vaccine harvests. Chabaud-riou et al. [31] have used G protein-based ELISA for the estimation of rabies viral antigen load in vaccines and for evaluating potency tests of the rabies vaccine. The ELISA was used as an in vitro tool to quantify the noncytopathic classical swine fever virus (CSFV) virus in the CSFV vaccine [32] in porcine kidney cell culture supernatants. Mantel et al. [33] have standardized a quantitative real-time RT-PCR system to evaluate the viral load of each serotype in the Yellow Fever and Chimeric Yellow Fever virus vaccine batch.

### Limitations of the Study

The current study shows that 0.08% formalin inactivated KFD vaccine is safe as well as immunologically potent in the mice model; however, the ability of 0.08% formalin used in the vaccine to reduce side effects of swelling and irritation at the site of vaccine inoculation needs to be examined in humans. Furthermore, future studies in vaccinated people are required to evaluate if the 0.08% formalin inactivated KFD vaccine retains the same immunological effectiveness as observed in mice in this study.

## 5. Conclusions

The study found that the use of 0.08% formalin completely inactivates KFDV in the vaccine and retains the immunogenicity and the potency to resist the virulent challenge. The reduction in formalin concentration to 0.08% in the vaccine may possibly reduce the pain, swelling, and other side effects due to formalin content at the site of vaccination in people. This eventually may increase the vaccine uptake and coverage aiding in the prevention and control of KFD disease in endemic areas before a new generation vaccine for KFD is made available.

Real-time PCR can be used for quantification of the noncytopathic viruses in the intermediate stages of the vaccine production process, and the real-time PCR results give an estimate of virus load. A final potency test of the vaccine can be performed in laboratory animals, only if the real-time PCR of individual harvests has the required CT value. This will save hundreds of mice that are used for quantification of virus load during intermediate stages of vaccine production.

## Figures and Tables

**Table 1:** Comparison of in vivo and in vitro titration of KFDV.

Virus Dilutions	% Infectivity	CT Values
10^−1^	100	22.05
10^−2^	100	24.13
10^−3^	100	26.09
10^−4^	100	29.54
10^−5^	100	31.18
10^−6^	100	34.02
10^−7^	20	-
10^−8^	0	-

## Data Availability

The data presented in this study are available on request from the corresponding author.
